# Omicron (B.1.1.529) BA.1 or BA.2-related effects on immune responses in previously naïve versus imprinted individuals: immune imprinting as an advantage in the humoral immune response against novel variants

**DOI:** 10.3389/fimmu.2023.1165769

**Published:** 2023-05-15

**Authors:** Sissy Therese Sonnleitner, Samira Walder, Ludwig Knabl, Roswitha Poernbacher, Thomas Tschurtschenthaler, Eva Hinterbichler, Stefanie Sonnleitner, Viktoria Muehlmann, Wilfried Posch, Gernot Walder

**Affiliations:** ^1^ Department of Virology, Medical Laboratory, Dr. Gernot Walder GmbH, Ausservillgraten, Austria; ^2^ Institute of Hygiene and Medical Microbiology, Medical University of Innsbruck, Innsbruck, Austria; ^3^ Tyrolpath Obrist Brunhuber GmbH, Zams, Austria

**Keywords:** SARS-CoV-2, omicron infection, immune response to omicron infection, serology, ELISpot, naïve versus imprinted, B.1.1.529

## Abstract

**Background:**

Immune imprinting is a phenomenon in which a person's immune system develops a specific immunological memory of the pathogen or vaccine due to a previous exposure. This memory basically leads to a faster and stronger immune response in a subsequent contact to the same pathogen or vaccine. However, what happens if the pathogen has changed considerably in the meantime due to mutations in the main target region of antibodies, as in the evolution of SARS-CoV-2 from the ancestral strain to B.1.1.529 (Omicron)? In this case, does immune imprinting also confer an advantage in repeated contact and does it lead to a stronger immune response?

**Methods:**

To clarify these questions, we investigated the effects of immune imprinting in the context of SARS-CoV-2 by comparing a group of previously infection-naïve versus imprinted study participants and determined differences in humoral and cellular immune responses during and after infection with strain SARS-CoV-2 B.1.1.529 BA.1 and BA.2, respectively. We used a commercial CLIA, immunoblots, IFN-γ ELISpots and a plaque-reduction neutralization test to generate a clear and comparable picture of the humoral and cellular immune response in the two study groups.

**Results:**

Imprinted participants developed significantly higher antibody titers and showed significantly stronger neutralization capacity against the ancestral strain, BA.1 and BA.5. The immune response of naïve study participants was narrower and related mainly to the receptor-binding domain, which resulted in a lower neutralization capacity against other strains including BA.5. Naïve study participants showed a significantly higher cellular immune response than the imprinted study group, indicating a higher antigenic challenge. The cellular immune response was directed against general structures of SARS-CoV-2 and not specifically against the receptor-binding domain.

**Conclusion:**

Viral variant infection elicits variant-specific antibodies and prior mRNA vaccination or infection with a previous SARS-CoV-2 variant imprints serological responses toward the ancestral strain rather than variant antigens. On the other hand, our study shows that the initially higher specific antibody titers due to former imprinting via vaccination or prior infection significantly increased the humoral immune response, and therefore outperformed the humoral immune response of naïve study participants.

## Introduction

Since the end of 2020, COVID-19 vaccines, in particular two messenger RNA (mRNA)-based COVID-19 vaccines, BNT162b2 (Comirnaty) from Biontech/Pfizer and mRNA-1273 from Moderna, have been authorized for use in the European Union. The vaccines have been intensively studied in both the development and surveillance phases, and dozens of studies have looked at the safety, efficacy, and tolerability of the vaccines (1–3). More than two years after its development, the target of the SARS-CoV-2 vaccine is still the spike (S) protein of the ancestral wild-type strain (Wuhan variant). This means that the immune system of the vaccinated is imprinted with this ancestral form of the spike protein.

Immunological imprinting, also known as immune imprinting, refers to a process in which an organism’s immune system is imprinted by a previous infection or exposure to a pathogen, e.g. by vaccination, basically resulting in an enhanced immune response to future infections with similar pathogens. With the emergence of new variants of SARS-CoV-2, there have been studies published that addressed this issue and found hints for antibody-dependent enhancement (ADE) in a few patients´ sera (4). In some cases, immune imprinting can make an organism more susceptible to a severe course of the disease. This phenomenon is known as ADE (5). This has been observed not only in dengue but also in zika virus infections, where prior infection with one dengue virus serotype may increase the risk of more severe dengue virus disease if a subsequent infection with a different dengue virus serotype occurs (6–9).

Since the first occurrence of SARS-CoV-2, however, a large number of SARS-CoV-2 variants have developed: In the course of the adaptation of SARS-CoV-2 to humans, Alpha (B.1.1.7) was the first variant of concern (VOC) to arise, whose modified spike (S) protein gave it a severe propagation advantage over the wild type strain “Wuhan” (10–12). In the study area of East Tyrol, VOC Alpha was followed by the Beta (B.1.351) and Gamma (P1) variants, and in early summer 2021, by Delta (B.1.617.2). The VOC Omicron (B.1.1.529) has dominated pandemic activity since early November 2021 and has now spread into a large number of subvariants (e.g. BA.1, BA.1.1, BA.2, BA.3, BA.4 and BA.5). Omicron has more than 30 non-silent mutations in the Spike protein (13–16) compared to the vaccine strain. 

What do these accumulations of mutations mean for future infections and the immune response? Is the immune imprinting on a considerably different ancestral variant now a disadvantage for the immune response against the Omicron variant and does ADE occur?

To pursue these questions, we recruited patients with acute infection with the then-current variant B.1.1.529 and determined the differences in the humoral and cellular immune response to an infection with B.1.1.529 between naïve patients and patients imprinted by previous infection or vaccination. For this purpose, B.1.1.529 BA.1 or BA.2-infected patients were followed over a period of three months. Titer movements of specific IgG antibodies against different targets on the virus surface were determined by CLIA and immunoblot. Furthermore, the specific cellular immune response was investigated by IFN-γ ELISpot. Finally, the neutralization capacity against the SARS-CoV-2 strains D614G, B.1.1.529 BA.1, and B.1.1.529 BA.5 was tested by plaque-reduction neutralization tests. The study shows significant differences in the humoral immune response between naïve and imprinted patients. In the case of SARS-CoV-2, immune imprinting seems to be an advantage even against new variants. Immune imprinting by vaccination or previous infection produces significantly higher antibody titers that serve a broader spectrum of targets. There is evidence that more severe courses of infection are linked to a reduced or non-concerted T and B cell response.

## Material & methods

### Study group

**Figure 1:**
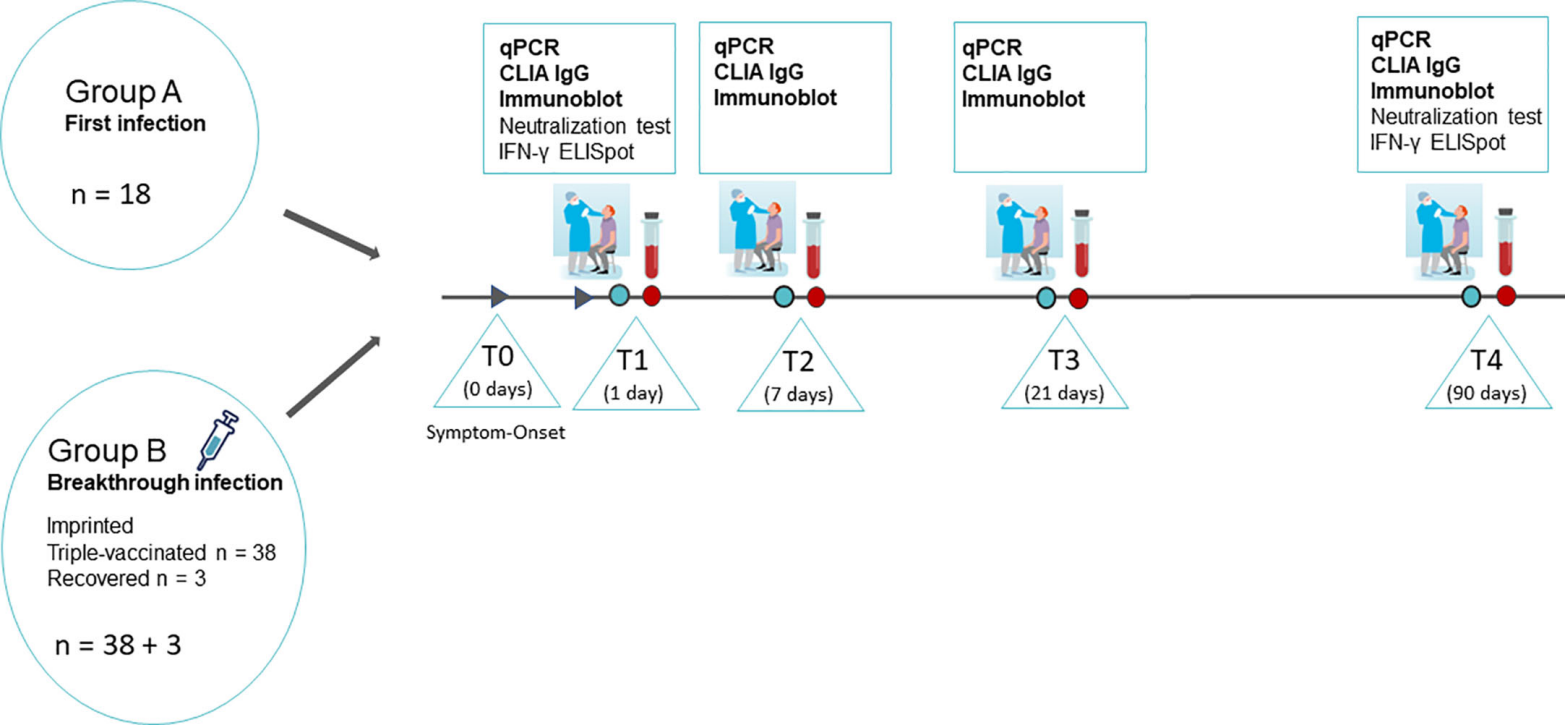
Overview of the study design. SARS-CoV-2 infected persons were recruited in the course of routine nasopharyngeal swabs diagnostics on the day of the onset of symptoms and the first positive PCR. The study participants were summoned for sample collection and medical supervision at T1 (no later than 1 day after the first positive PCR), T2 (1 week after T1), T3 (3 weeks after T1), and T4 (3 months after T1). Nasopharyngeal swabs and full blood samples were taken at all four time points. Additionally, 24 mL of peripheral blood in lithium-heparin tubes were sampled on T1 and T4 for isolation of PMBCs and subsequent IFN-γ ELISpot. Graphs partially provided by https://de.freepik.com/search?format=search&query=corona%20nasenabstrich.

SARS-CoV-2- specific PCR- positive patients were recruited for participation in the serological study in the course of routine nasopharyngeal swabs from January 2022 to April 2022. Informed consent was given by each participant, and anamnesis, symptoms, and the course of the disease were recorded by personal interview, and blood sampling and smear collection were obtained at T1 (no later than 1 day after the first positive PCR), T2 (1 week after T1), T3 (3 weeks after T1), and T4 (3 months after T1). An overview describing the study design is given in **Figure 1**.

The severity of the disease for each patient was assessed using a standardized questionnaire including age, sex, preexisting as well as acute physical condition and rated as a mild, moderate, or severe course of disease according to the definitions previously published (17). Briefly, in this study, we described a course as “mild” if the patient self-reported being mildly ill (e.g., fever, cough, sore throat, malaise, headache, muscle pain, nausea, vomiting, diarrhea, and loss of taste and smell) but who did not have shortness of breath or dyspnea; “moderate” if there were limitations in performing activities of daily living and/or subjective feelings of being ill and if the oxygen saturation measured by pulse oximetry (SpO2) was ≥ 94% on room air at sea level, and “severe” if the patient had to seek medical attention and/or needed to take medication to relieve symptoms and/or was bedridden and had SpO2 30 breaths/min or lung infiltrates >50%.

### RT-qPCR and melting curve analysis

Nasopharyngeal swabs were taken in a standardized way in home quarantine in the context of primary care by a study nurse or physician. The patient gave full written consent for the case to be attended and published and the study was performed according to the principles of the Declaration of Helsinki 2013. The swabs were analyzed by RT-qPCR for the presence of SARS-CoV-2 specific RNA (Simplexa COVID-19 Direct Kit, DiaSorin Molecular, Italy), and the Omicron strain was typed by melting curve analysis using primers targeting the SARS Spike the del69-70 deletion and the S371L, S373P mutations (TIB Molbiol GmbH, Germany) according to the manufacturer´s protocols. The results of the melting curve analysis were randomly verified by whole genome sequencing.

### Whole genome sequencing

Libraries were prepared according to the Ion AmpliSeq SARS-CoV-2 Research Panel (Thermofisher, USA), and library construction and sequencing protocol with the Library Kit Plus (Thermo Fisher Scientific, Waltham, Massachusetts, USA; Cat. No. 4488990). The Amplicons were cleaned up with AMPure XP beads (Beckman Coulter, Germany) in a 1:1 ratio. The libraries were quantified using the Ion Library TaqMan Quantitation Kit (Cat. No. 4468802), and normalizing, pooling and sequencing were performed using an Ion Torrent S5 Plus. Ion Torrent Suite software (v 5.12.2) of the Ion S5 sequencer was used to map the generated reads to a SARS-CoV-2 reference genome (Wuhan-Hu-1; GenBank accession numbers NC_045512 and MN908947.3), using TMAP software included in the Torrent Suite, and sequences with reads lower than 1 x 10^6^ were excluded. The following plugins were used: Coverage Analysis (v5.10.0.3), Variant Caller (v.5.12.04) for mutation calls both with “Generic—S5/S5XL (510/520/530)—Somatic—Low Stringency” and “Generic‐S5/S5XL (510/520/530)‐Germ Line‐Low Stringency” default parameters and COVID19AnnotateSnpEff (v.1.0.), a plugin specifically developed for SARS‐CoV‐2 that can predict the effect of base substitution. No ultra-deep sequencing was performed and only mutations visible in the stated analysis methods were listed and rated. FASTA files containing the raw reads were inspected for quality criteria (mapped, targeted, filtered reads, mean depth, and uniformity) using Thermofisher Software. Multiple sequence alignments were performed using Unipro UGENE (18) as well as MEGA X (19). The SARS-CoV-2 genomes were compared to the reference NC 045512.2-Wuhan-Hu-1. Viral genome assembly and screening for distinct mutations were performed online using nextstrain.org (https://github.com/nextstrain/ncov/blob/master/defaults/clades.tsv;chttps://clades.nextstrain.org/). The identification of pangolin lineages was carried out using Pangolin software, v.2.4.2. (https://pangolin.cog-uk.io/). The whole genome sequences are deposited at Genbank under the accession numbers OQ520264.

### Serology

Serological tests were performed using the LIAISON SARS-CoV-2 TrimericS IgG (DiaSorin S.p.A., Saluggia, Italy) (LIAISON) and a commercial immunoblot (ViraChip assay by Viramed, Munich, Germany).

### Microarray immunoblot

The ViraChip assay detects temporal antibody profiles of different immunoglobulin classes against the purified surface proteins S1 (Wuhan), RBD (Wuhan), RBDo (Omicron), RBDd (Delta), and S2 (Wuhan) as well as the nucleocapsid protein N (Wuhan) of SARS-CoV-2 as antigens in a commercial, miniaturized 96 wells protein microarray. The quantitative antibody measurement in BAU/mL was performed on a ViraChip Scanner using ViraChip Software.

### CLIA SARS-CoV-2 TrimericS IgG

IgG antibodies reactive with the spike protein (S1/S2 domain) were determined using a commercially available chemiluminescent immunoassay (CLIA; LIAISON SARS-CoV-2 TrimericS IgG). The assay was performed on the LIAISON XL Analyzer according to the manufacturer’s instructions and yielded the binding antibody units per mL (BAU/mL) according to the WHO International Standards for the Anti-SARS-CoV-2-immunoglobulin-binding activity (NIBSC 20-136).

### Isolation of SARS‐CoV‐2

The isolation of SARS‐CoV‐2 strains wildtype (WT, D614G), Omicron BA.1, and Omicron BA.5 was attempted from RT‐qPCR positive nasopharyngeal swabs by inoculation on VeroB4 (no. ACC‐33, DSMZ) in T25 tissue culture flasks for 1 h at 35°C. After incubation, the sample was removed and Medium199 (Lonza, Switzerland) with 2.5% fetal calf serum (FCS; Lonza, Switzerland) and a mixture of antibiotics (streptomycin, vancomycin, and penicillin, each 1 µg/ml) was added. Virus cultures were monitored daily for cytopathic effects and tested for specific viral RNA after 1 week. Isolation was considered successful when the cytopathic effect was 80% – 100% in passage 0 as well as passage 1 and/or the Ct value in RT-qPCR was lower than 20. Highly positive supernatants were harvested, centrifuged at 3,400 *g* for 5 min, and stored at −80°C in Medium199 with 10% FCS. A further passage of diverse isolates was performed to obtain the highest possible concentration, which was Ct 11 on average. All work involving infectious SARS‐CoV‐2 was carried out in a BSL3 facility, following the institutional guidelines and regulations. The identification of the strains was performed *via* next-generation sequencing.

### Virus titration for the quantification in plaque-forming units

VeroB4 cells (ACC-33, DSMZ) were seeded in flat-bottom 96 well plates with Medium199 (Lonza, Switzerland) and 10% fetal calf serum (Lonza, Switzerland) at a density of about 10^6^ cells/ml to give a confluent monolayer. On the next day, an infectivity titration was carried out to determine 100 tissue culture infectious doses of 50% (100 TCID_50_) (20, 21).

### PBMC isolation and IFN-γ ELISpots

Peripheral blood mononuclear cells (PBMCs) were isolated using SepMat-50-Tubes (Stemcell Technologies, Canada), and filled with a 15 mL Lymphoprep density gradient medium (Stemcell Technologies, Canada). Each tube was filled with 12.5 mL lithium-heparin blood (Vacuette, Greiner bio-one, Austria) 1:1 mixed with phosphate-buffered saline (PBS) and centrifugated for 10 min at 1,200 g with 3.4 max acceleration and 4/10 max braking. The PBMCs were isolated, briefly transferred into a new 50 mL tube, and washed twice with PBS. The PBMC pellet was resuspended in Cryostor CS10-Medium (Stemcell Technologies, Canada) and frozen at -80°C using freeze racks filled with 70% isopropanol until further use (Mr. Frosty, Thermo Scientific, USA) at a density of 3 x 10^6^ cells/mL.

The ELISpot assay was performed using a commercially available pre-coated human SARS-CoV-2-specific IFN-γ ELISpot kit according to the manufacturer´s protocol (AutoImmun Diagnostika, GmbH, Germany; Cat.no. ELSP 5500). To start the ELISpot, PBMCs were thawed, and the cryomedium was quickly replaced by x-vivo (X-VIVO TM-10 Serum-free hematopoietic cell medium; BEBP02-055Q, Lonza, Switzerland), and the cells were counted. A total of 2 × 10^5^ PBMCs were incubated in duplicate with x-vivo as a negative control, pokeweed mitogen as a positive control, and 15–20mer peptide pools for SARS-CoV-2 Wuhan (S-, N-, M- and E-region) and RBD of SARS-CoV-2 strain Omicron (AutoImmun Diagnostika GmbH, Germany) as specific antigenic peptide pools. After incubation at 37°C for 20 h in a sterile and humidified atmosphere, plates were washed with a washing buffer (AutoImmun Diagnostika GmbH, Germany) and stained with the kit-specific reagents according to the manufacturer’s protocol. Plates were then washed several times under running water and dried overnight. Spot forming units (SFU)/100,000 cells were counted using an automated AID ELISpot reader system (AutoImmun Diagnostika GmbH, Germany).

The assessment criteria for the ELISpots were a minimum of 50 SFU in the positive control and a maximum of 10 SFU in the negative control according to the manufacturer´s definitions. When those criteria were fulfilled, the stimulation index (SI) was calculated by dividing the mean SFU numbers in the antigen-specific wells by the mean SFU numbers of the negative control. The test was assessed as negative when there was an SI < 2, according to a previous determination of the cutoff by well-defined pre-pandemic PBMC samples and by PBMCs from SARS-CoV-2-naïve individuals. The test was suggested to be poorly reactive when there was an SI between 2 and 3 and to be reactive when there was an SI ≥ 3, as defined by the manufacturer. According to standardized laboratory procedures, in each assay, a standard laboratory control sample of a high-reactive and a non-reactive PBMC sample, respectively, were run to determine inter-assay variations. Only assays with less than two standard deviations of the high-reactive and the non-reactive PBMC control sample, respectively, were defined as valid.

### Plaque-reduction neutralization tests

The neutralization ability of antibodies was determined by performing a plaque-reduction neutralization test. VeroB4 cells (ACC-33, DSMZ) were seeded in flat-bottom 96 well plates (Sarstedt, Germany) with Medium199 (Lonza, Switzerland) and 10% FCS (Lonza, Switzerland) at a density of approximately 10^6^ cells/ml to give a confluent monolayer. On the next day, an infectivity titration was carried out to determine 100 tissue culture infectious doses of 50% (100 TCID50) (20). Patients´ sera were heat inactivated by incubation at 56°C for 30 min and titrated in duplicate at an initial dilution titer of 1:4 in Medium199 containing 3% fetal calf serum. Equal volumes of virus (1 × 10^5^ TCID_50_) and serum dilutions in Medium199 were mixed and subsequently incubated for 1 h at 35°C in U-bottom 96-well plates (Thermo Scientific Nunc, USA). After incubation, a pre-seeded flat-bottom 96-well plate with confluent VeroB4 cells was used, the medium was discarded, the incubated mixture of the patient´s serum and defined virus solution was transferred to each corresponding well of the flat-bottom plate, and the plate was incubated for 96 h at 35°C. Incubation was stopped by discarding the supernatant, cells were washed twice in PBS, fixed with paraformaldehyde 4%, and dyed with crystal violet. All steps were performed under strict observation and in compliance with biosafety level 3. The analysis was carried out using specially programmed software for the AID ELISpot reader system (AutoImmun Diagnostika GmbH, Germany) by counting the percentage of cytopathic effects in each well.

### Statistics

Dichotomous data were evaluated by a chi-squared test or Fisher´s exact test in the case of small group size (*n* < 60). A two-sided significance level of *p* < 0.05 was used for determining statistical significance. After testing for distribution (Kolmogorov–Smirnov-test), non-parametric continuous independent variables were compared using the Mann–Whitney-*U* test for each time point. Dependent non-parametric variables were compared using the Wilcoxon-rank test. All statistical analyses were performed with SPSS Version 23.0 (Chicago, IL, USA).

## Results

### Characteristics of the study group

The study group consisted of 59 volunteers who tested positive for SARS-CoV-2, strain Omicron in the course of routine testing between January to April 2022. The average age of the study participants was 49.1 years, and the age structure in the study was not normally distributed (*p* = 0.009). An overview of the study group concerning sex, age group, and initial serological status is given in **Table 1**. Further information on the study group can be found in anonymized form in the **Supplementary material**.

**Table 1:** Overview of the study participants´ initial serostatus, tested *via* microarray immunoblot (ViraChip assay, Viramed, Munich, Germany) at time point 1 with regard to age group, sex, and previous immune status against SARS-CoV-2.

female	initial serostatus			
age group	naïve	[%]	imprinted	[%]
21-30	2	5.9	3	8.8
31-40	3	8.8	8	23.5
41-50	0	0.0	2	5.9
51-60	3	8.8	5	14.7
61-70	3	8.8	3	8.8
71-80	0	0.0	2	5.9
	11	32.4	23	67.6

male	initial serostatus			
age group	naïve	[%]	imprinted	[%]
21-30	0	0.0	1	4.0
31-40	1	4.0	3	12.0
41-50	2	8.0	5	20.0
51-60	4	16.0	3	12.0
61-70	0	0.0	3	12.0
71-80	0	0.0	3	12.0
	7	28.0	18	72.0

The group consisted of 34 women and 25 men between 21 and 80 years of age. During medical supervision, each patient´s history of immunization or infection with SARS-CoV-2 was recorded and symptoms as well as the severity of the Omicron infection were assessed on-site. 87.1% of the vaccinated patients had received three doses (27/31). The survey showed that the immune status reported by the study participants did not necessarily correspond to the serological results, as shown in **Table 2**. All participants who reported being vaccinated or having recovered from a SARS-CoV-2 infection showed high titers of specific IgG antibodies. However, 18.2% of the study participants who claimed to be unvaccinated and did not report any previous infection had specific antibodies (4/22). Those four patients were counted in the imprinted study group and we, therefore, decided not to divide the study participants into groups of vaccinated and unvaccinated, but into groups with and without an initial SARS-CoV-2 specific immune response, and we will refer to these cohorts as (infection- and vaccination-)naïve versus imprinted study participants. The category of imprinted study participants comprised 38 partially or fully vaccinated and three infection-recovered individuals. Pfizer BioNTech COVID-19 vaccine (BNT162b2) was used most frequently for immunization (252.6%; 20/38), followed by Moderna COVID-19 vaccine (mRNA-1273) (26.3%; 10/38), AstraZeneca (ChAdOx1 nCov-19) (13.2%; 5/38), and Johnson & Johnson (Jcovden) (7.9%; 3/38). Given the reported time of infection (November 2020 and November 2021, respectively), two of the three recovered persons had been infected with D614G and one with the Delta variant (B.1.617.2).

**Table 2:** Calculation of the agreement between the personal assessment of the 59 study participants about their own initial immune status and the results of humoral and cellular diagnostics about it, calculated as - positive predictive value (PPV).

		Humoral immunity					Cell-mediated immunity					
		positive	[%]	negative	[%]	PPV	total	positive	[%]	negative	[%]	PPV
**vaccinated**	28	58	100.0	0	0.0	100.0	29	12	52.2	11	47.8	52.2
**recovered**	3	3	100.0	0	0.0	100.0	3	2	66.7	1	33.3	33.33
**not imprinted**	28	4	14.3	24	85.7	85.7	23	10	30.8	19	69.2	69.2
**total**	59	35	24	24			55	24		31		

A closer look at the specific cellular immune status of the study participants revealed that only 58.1% of the vaccinated participants showed a positive result in the SARS-CoV-2 specific IFN-γ ELISpot (18/31) and 0% of the unvaccinated study participants without proven previous encounter with SARS-CoV-2 tested positive in the SARS-CoV-2 specific IFN-γ ELISpot (0/18). Since the study aims to clarify the influence of immune imprinting on the development of the immunological response upon re-contact, the patients were divided into two groups according to their respective initial humoral or cellular immune status: (infection- and vaccination-) naïve versus imprinted.

### SARS-CoV-2 strains and viral loads in this study

In total, the strain type of 54 of the 59 study participants (91.5%) could be determined using melting curve analysis. The specific strain identification was confirmed in 27 cases *via* next-generation sequencing. The whole genome sequences were deposited at Genbank under the accession number OQ520264. In 62.7% of the cases, BA.1 was identified as the causative agent, while in 28.8% (37/59) of the cases, it was BA.2 (17/59). In 8.5% (5 of 59) of nasopharyngeal swabs from T1, the closer identification of the Omicron strain remained unclear. There was no significant difference in the course of disease between BA.1 and BA.2.

**Figure 2:**
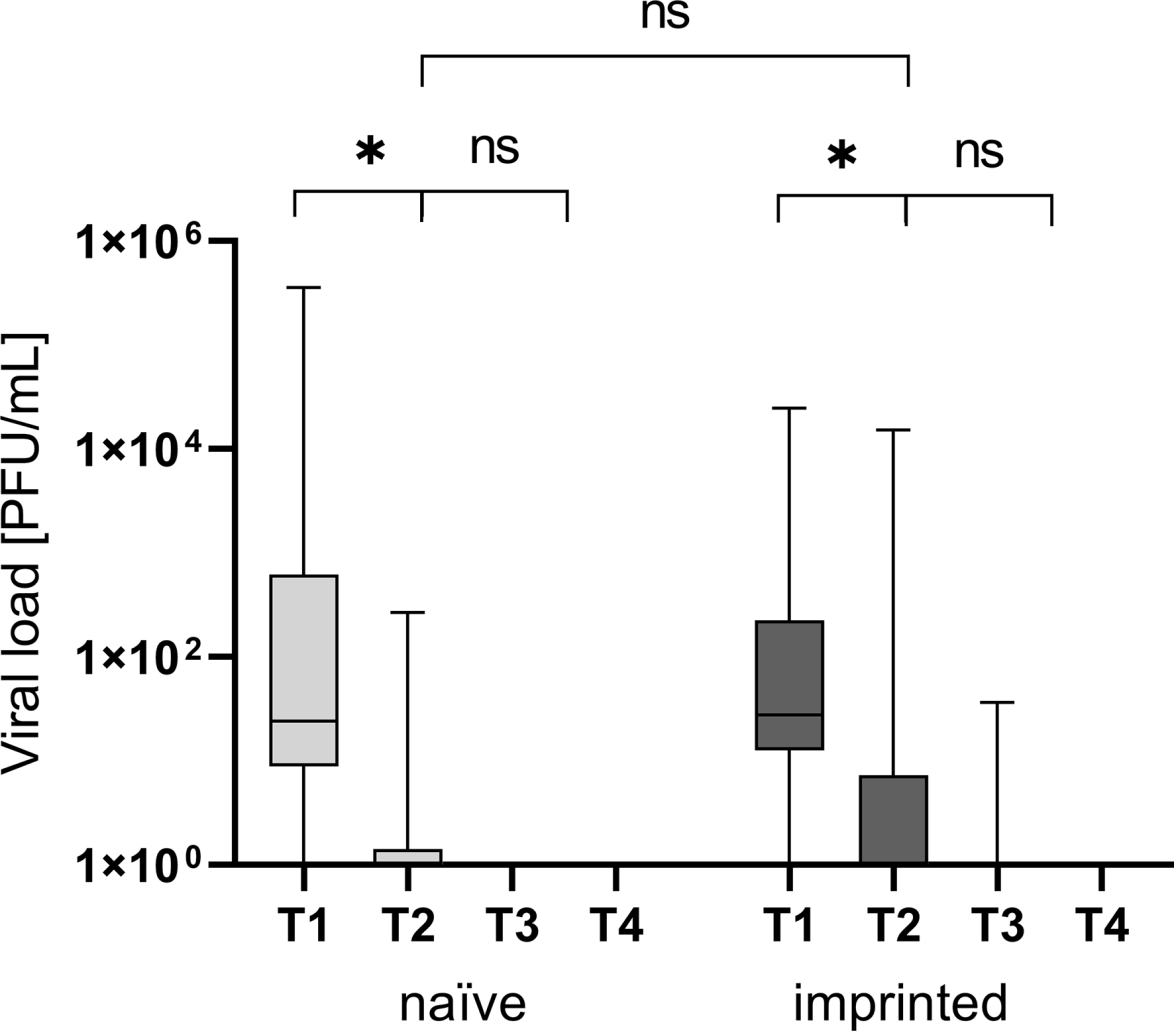
Viral load in plaque-forming units/mL at the different time points T1 (one day after the first positive PCR result), T2 (one week after T1), T3 (three weeks after T1), and T4 (three months after T1) in the different groups of study participants divided into those who were initially naïve (n = 18) versus those who were initially immunized (n = 41). ns - not significant; *p < 0.05.

At time point 2, one week after the onset of the symptoms, the number of PCR-positive patients was insignificantly higher in the naïve study group (87.5 versus 71.4%; x^2^ = 2.14), as shown in **Figure 2**.

The naïve study group had a higher nasopharyngeal viral load at T1 (1 day after the first positive PCR result)(22,023 versus 1,369 PFU/mL; p > 0.05). Neither the differences in the nasopharyngeal viral load were significant at T1 nor was the decrease of viral load and therefore infectivity between the two study groups (*p* > 0.05; **Figure 2**).

In the imprinted study group, the nasopharyngeal viral load decreased by 69.3% within seven days from 1,369 PFU/mL to an average of 421 PFU/mL, and one patient (4.5%, 1/22) was still positive after three weeks (T3) with a low viral load. In the naïve study group, the viral load decreased by 99.9% from 22,023 PFU/mL to 16 PFU/mL within seven days. The decrease of the viral load between T1 and T2 was significant in both groups (*p* < 0.05; *p* = 0.009 and 0.008, respectively).

### Humoral immune response – an infection with Omicron leads to a significant increase of specific IgG antibodies

**Figure 3:**
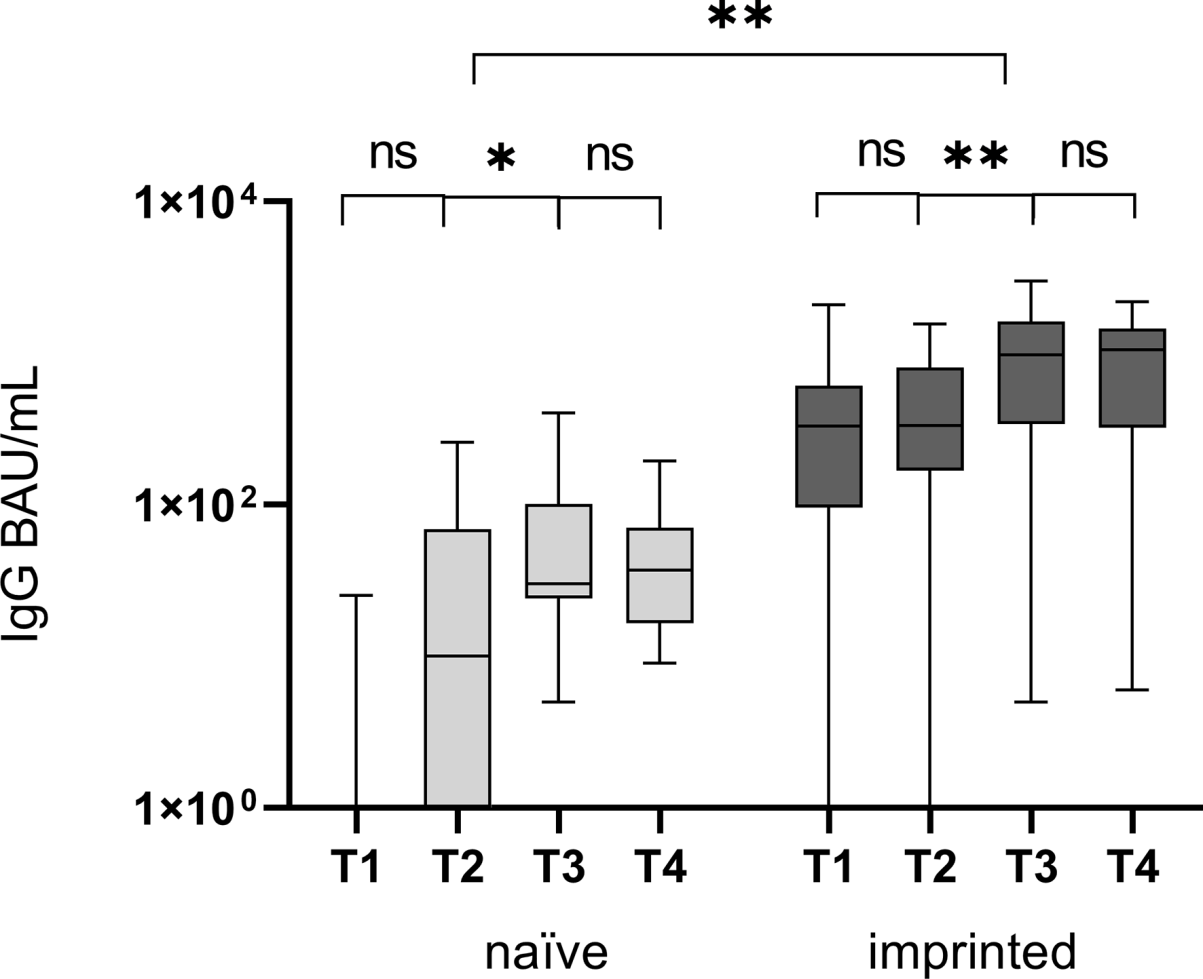
Comparison of specific IgG antibody titers, determined by CLIA, at four different time points T1 (one day after the first positive PCR result), T2 (one week after T1), T3 (three weeks after T1), and T4 (three months after T1) in the groups of study participants without initial specific antibodies (naïve) versus those with initial specific antibodies (immunized). The Omicron infection caused a significant increase in specific IgG antibodies in both groups between T2 and T3, after the first week and before the end of the third week. ns - no significant difference; *p < 0.05; **p > 0.01.

There was a significantly higher level of specific IgG antibodies at T1 and T4 in the imprinted group (mean T1: 493.7 BAU/mL, mean T4: 952.7 BAU/mL; *p* < 0.001) compared to the naïve study group (mean T1: 3.2 BAU/mL, mean T4: 51.2 BAU/mL; *p* = 0.004) (**Figure 3**). The increase from T2 to T3 was significant in both groups (p < 0.001). Specific IgM antibodies could not be detected by CLIA, neither at T1 nor at T2. The highest specific IgG antibody titers at the beginning of the Omicron infection in the immunized study group were above 1,500 BAU/mL (n = 3), with one not even above 2,000 BAU/mL (n = 1). In both groups, the specific antibody titer was highest at T3 (91.7 and 1,016.3 BAU/mL) and decreased by 45% and 6.2% at T4, three months after symptom onset (51.2 and 952.7 BAU/mL).

**Figure 4:**
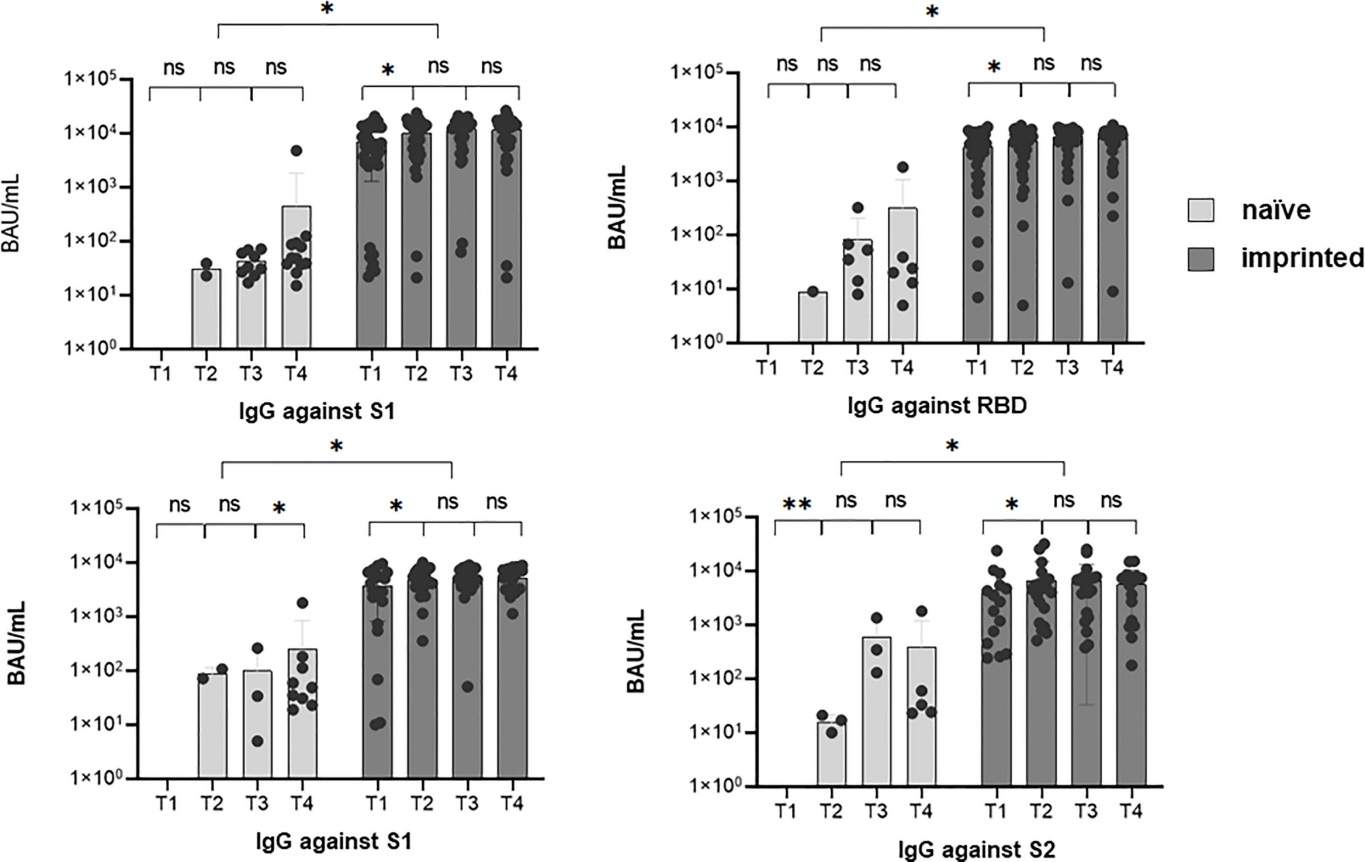
The development of specific IgG antibodies against A) spike 1 (S1), B) spike 2 (S2), C) receptor-binding domain (RBD), and D) nucleocapsid at four different sampling time points T1 (1 day after the first positive PCR) to T4 (3 months after the first positive PCR) during an existing Omicron infection, in two different groups of study participants, determined by a microarray immunoblot (Viramed, Germany). One group was previously naïve, without former contact with SARS-CoV-2, the other group was previously imprinted due to vaccination or natural infection with a strain other than Omicron. The increases in specific IgG against all four targets were significant from T1 to T2 (*p* < 0.05) in the imprinted study group and for the targets N and S2 in the naïve study group. ns - no significant difference; *p < 0.05; **p > 0.01.

We further determined the epitopes targeted by the specific IgG antibodies in the course of the Omicron infection. For this purpose, the ViraChip assay was used, which carries the purified surface proteins spike 1 (S1) (Wuhan), receptor-binding domain (RBD) (Wuhan), RBDo (Omicron), RBDd (Delta), and spike 2 (S2) (Wuhan) as well as the nucleocapsid protein N (Wuhan) of SARS-CoV-2 as antigens and thus enables a quantitative differentiation of the IgG antibodies. **Figure 4** compares the development of specific IgG antibodies over the four time points T1 to T4 in the two study groups of initially immunized versus initially naïve patients.

Overall, the IgG antibody titers were significantly higher in the study group of the initially immunized patients (*p* < 0.05). The immunized patients showed significant increases against all four investigated targets, S1, S2, RBD (Wuhan), and N, within the first weeks of infection (*p* < 0.05). In contrast, in immunologically naïve patients, specific IgG antibodies against the targets S2 and N significantly increased, but not against S1 and wildtype-specific RBD.

**Figure 5:**
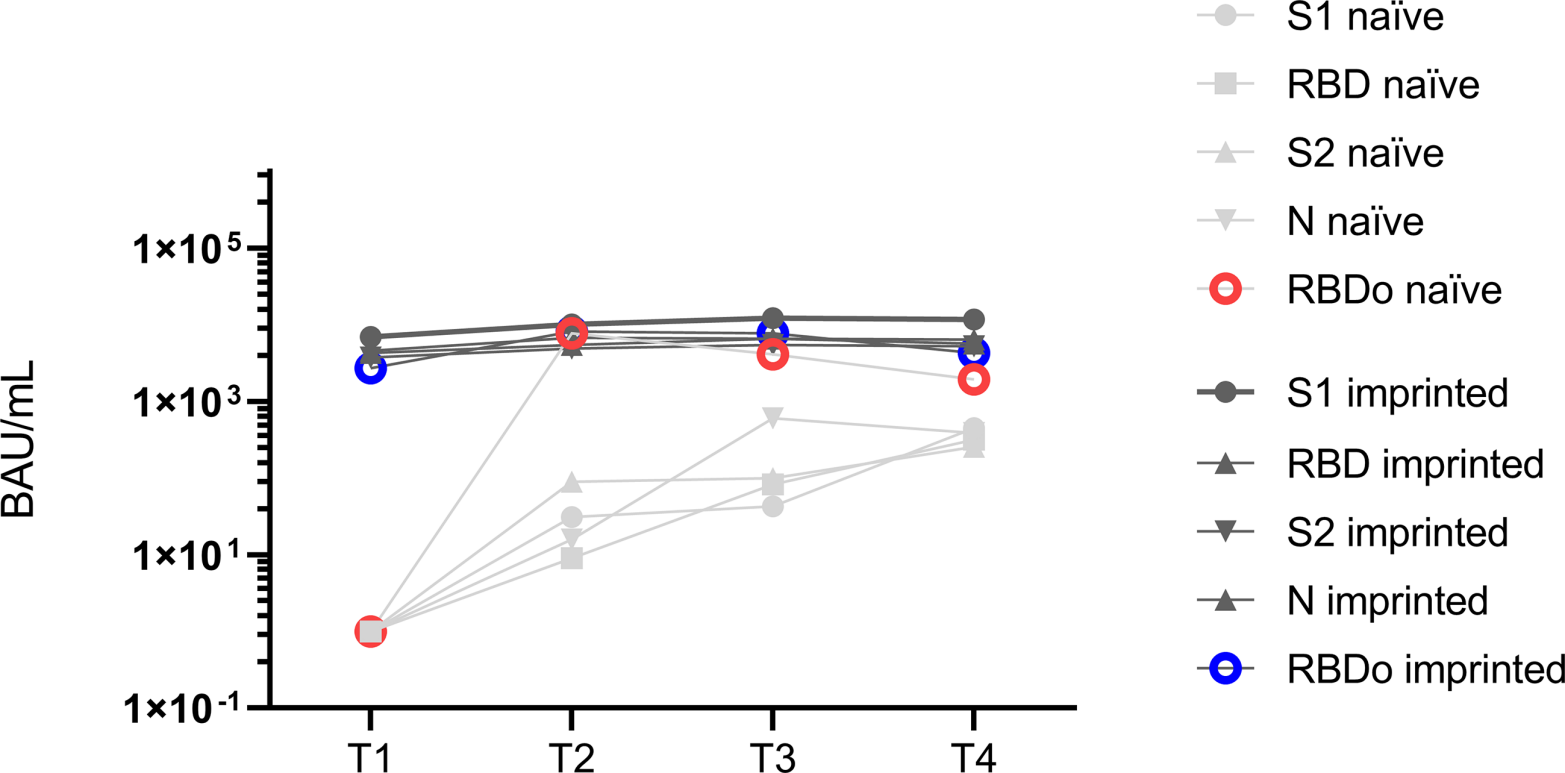
Mean values of specific IgG antibody titers against the different targets spike 1 (S1), spike 2 (S2), receptor-binding domain (RBD), nucleocapsid (N), and Omicron-specific receptor-binding domain (RBDo) in two comparative groups of immunologically naïve and imprinted (immunized) study participants.

Evaluating the antibody development against the Omicron-specific RBD (RBDo) revealed significant increases in specific IgG antibodies against this target in the initially naïve study group, as shown in **Figure 5** (*p* = 0.003).

The immunized study patients showed significantly higher increases in specific antibodies against the targets: S1 (*p* < 0.001), N (*p* < 0.016), and RBD Wuhan (*p* < 0.007). However, it was striking that the naïve study participants formed specific IgG antibodies specifically against one target region, namely, against the Omicron-specific receptor-binding domain (RBDo) (*p* < 0.05).

### T cell-mediated immune response – an infection with Omicron leads to a significant increase in specific IFN-γ -response in naïve patients

#### A cellular specification to Omicron is observed only in naïve patients

The specific cellular immune response of the study participants was measured using IFN-γ ELISpot at T1 (1 day after the first positive PCR) and T4 (3 months after the first positive PCR). The study group was again divided into naïve and imprinted, regarding the T-cellular immune response at T1.

To determine a cellular immune response to the Omicron-specific RBD (RBDo) in the course of an infection with Omicron, the PBMCs were incubated in the ELISpot with Omicron-specific RBD peptide pools.

Using a SARS-CoV-2 peptide pool derived from the Wuhan-strain (wildtype), the initial SI was 1.0 in the naïve group and 4.9 in the imprinted group, yielding an insignificant rise in the naïve group (from mean 1.0 SI to1.8 SI; p > 0.05) as well as in the imprinted group (from mean 4.9 SI vs 6.8 SI; p > 0.05).

**Figure 6:**
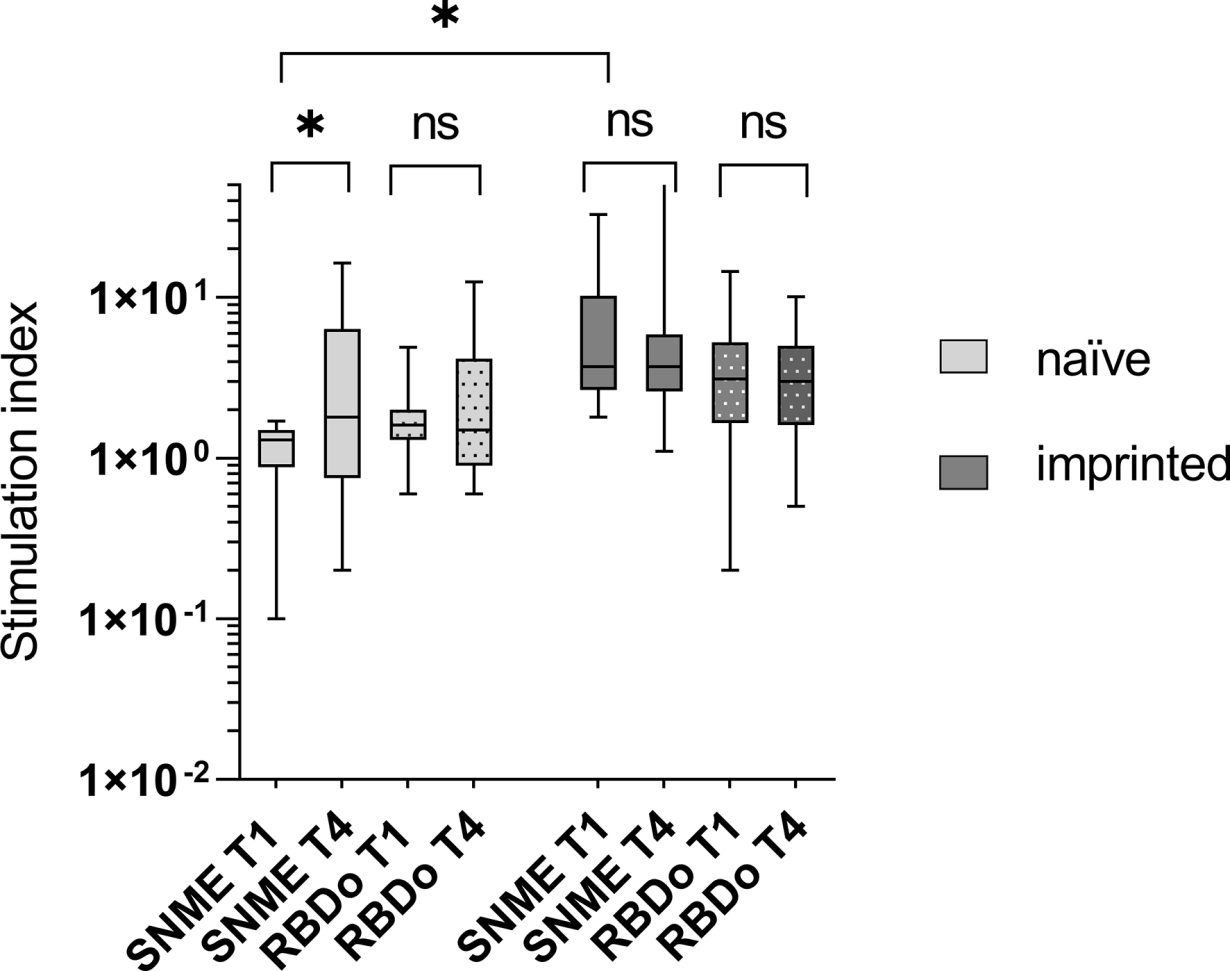
Visualization of the development of the cellular immune response against SARS-CoV-2 wildtype peptide pools and Omicron-specific RBD peptide pools in the two different study groups of naïve versus initially positive study participants. The cellular immune response was determined using IFN-γ ELISpot. WT – peptide pools combining S, N, M, and E regions of SARS-CoV-2 wildtype (Wuhan); RBDo – peptide pools of the Omicron-specific receptor-binding domain. ns - not significant; *p < 0.05.

Using Omicron-specific peptide pools consisting of the receptor-binding domain (RBDo) gave a similar result: both groups showed insignificant increases in IFN-γ ELISpot during the course of the infection. The SI remained constant in both groups (SI 1.8 to 1.9 in the naïve and 3.2 to 3.3 in the imprinted study group; *p* > 0.05). A visualization of the development of the cellular immune response against SARS-CoV-2 wildtype peptide pools and Omicron-specific RBD peptide pools in the two different study groups is given in **Figure 6**.

The stimulation index was significantly higher against both the Wuhan-specific peptide pool SNME and the omicron-specific peptide pool RBDo in the group of imprinted study participants than in the group of naïve study participants (p = 0.003 and 0.03, respectively).

### BA.2 is associated with insignificantly more severe symptoms

In 91.5% of the cases, the strain could be identified via melting curve analysis (54/59). A total of 37 nasopharyngeal swabs from T1 could be assigned to Omicron BA.1 (62.7%), while 17 were identified as Omicron BA.2 (28.8%), and the strain could not be determined in four swabs (6.8%).

**Figure 7:**
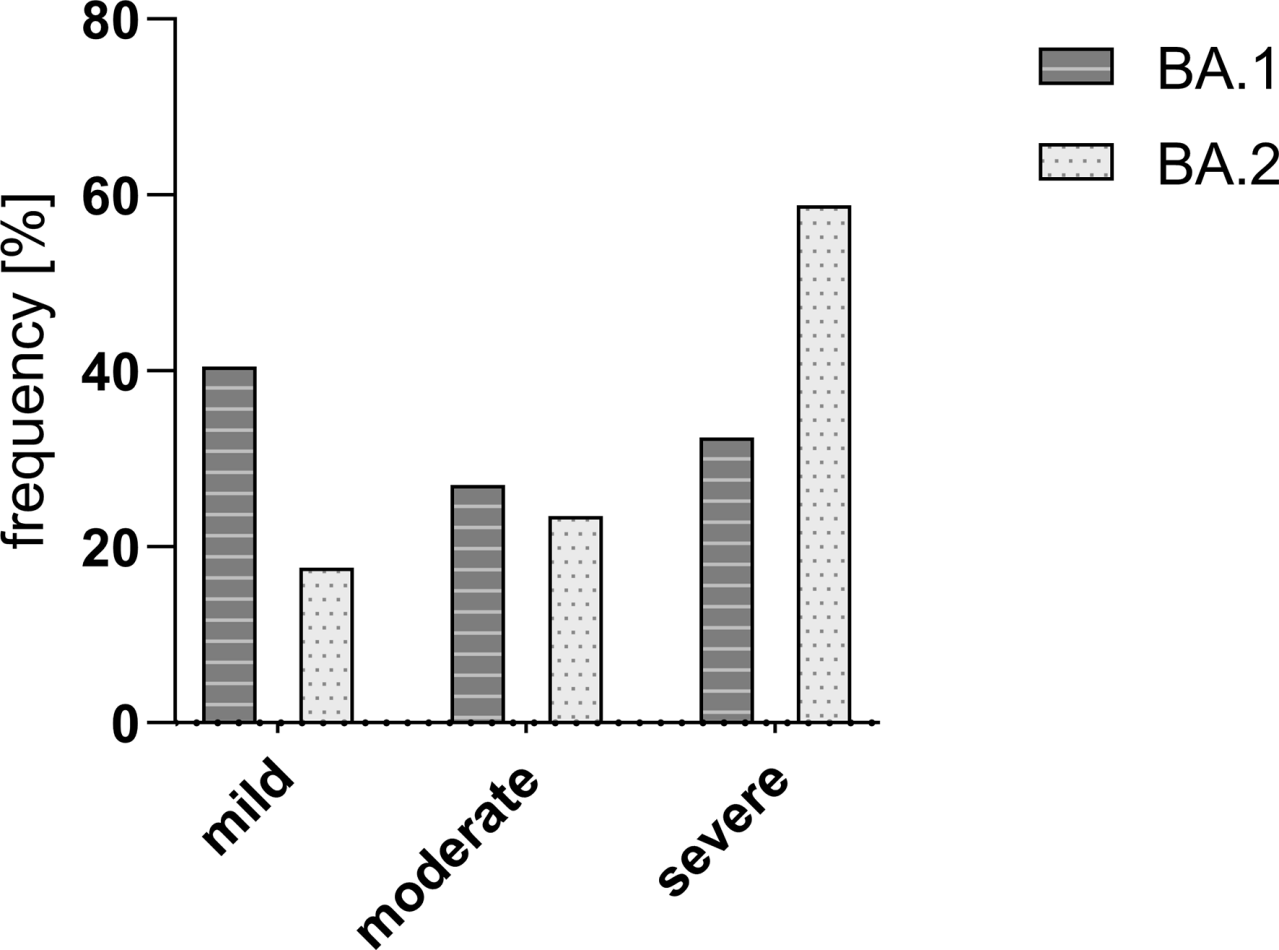
Frequency [%] of Omicron strains BA.1 and BA.2 in the study group divided into different courses of disease. There was no significant difference in the severity between the two compared SARS-CoV-2 strains BA.1 and BA.2. Mild – mild course of the disease; moderate – moderate course of the disease; and severe – severe course of the disease.

The majority of BA.1 cases reported a mild course of infection (24/59; 40.5%) whereas the majority of BA.2 experienced severe symptoms (58.8%; p > 0.05). An overview of the percent distribution of strains BA.1 and BA.2 over the category severity is shown in **Figure 7**.

There was no significant difference in the viral load at any of the four time points and the initial viral load (*p* > 0.05). No correlation was found between the initial viral load and the severity of the course (*k* = 0.059). There was also no correlation between the initial viral load and the initial specific antibody titer (*k* = 0.183).

### A rapid increase in specific antibodies is associated with a less severe course of the disease

Out of the 59 study participants, 18 had no detectable specific IgG antibodies in the immunoblot at the onset of the Omicron infection and were rated as “naïve”. The proportion of patients with a severe course was higher in the naïve group (9/18; 50% versus 26/59; 44.1%), but the difference was not significant (*p* > 0.05).

**Figure 8:**
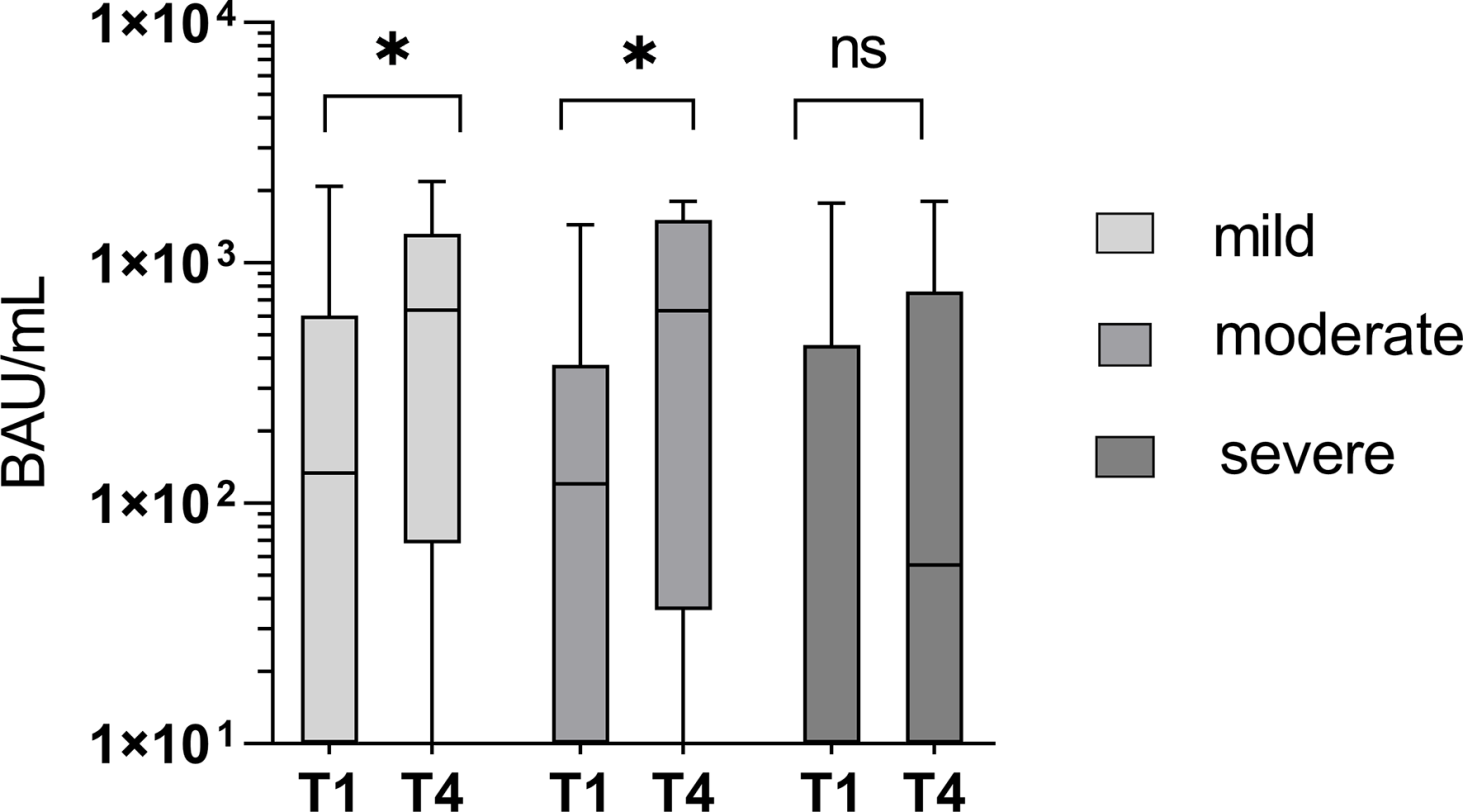
Significant increase in specific IgG antibody titers between T1 and T4 in mild and moderate courses of the disease (p = 0.006 and 0.011), but not in severe courses of the disease (p = 0.131). ns - not significant; *p < 0.05.

However, a significant increase in specific IgG antibodies between T1 and T4 was associated with mild (T1: 690.8 BAU/mL; T4: 973.2 BAU/mL; *p* = 0.006; n = 14) and moderate courses (T1: 485.4BAU/mL; T4: 990.19 BAU/mL; *p* = 0.011; n = 13), but not in patients suffering a severe course (T1: 728.6 BAU/mL; T4: 706.1 BAU/mL; *p* = 0.131; n = 26). The overall mean titer of specific IgG antibodies was not significantly different between the groups (p > 0.05). **Figure 8** shows the course of specific IgG antibodies between T1 and T4 in the three severity categories: “mild”, “moderate”, and “severe”.

A moderate correlation (r = 0.369) was found between the course of the disease and the initial RBDo-specific IgG titer as well as a moderate negative correlation (r = -0.340) between the course of the disease and the initial specific IgG titer against the target N.

### Specific T-cell response has little influence on the severity of infection

**Figure 9:**
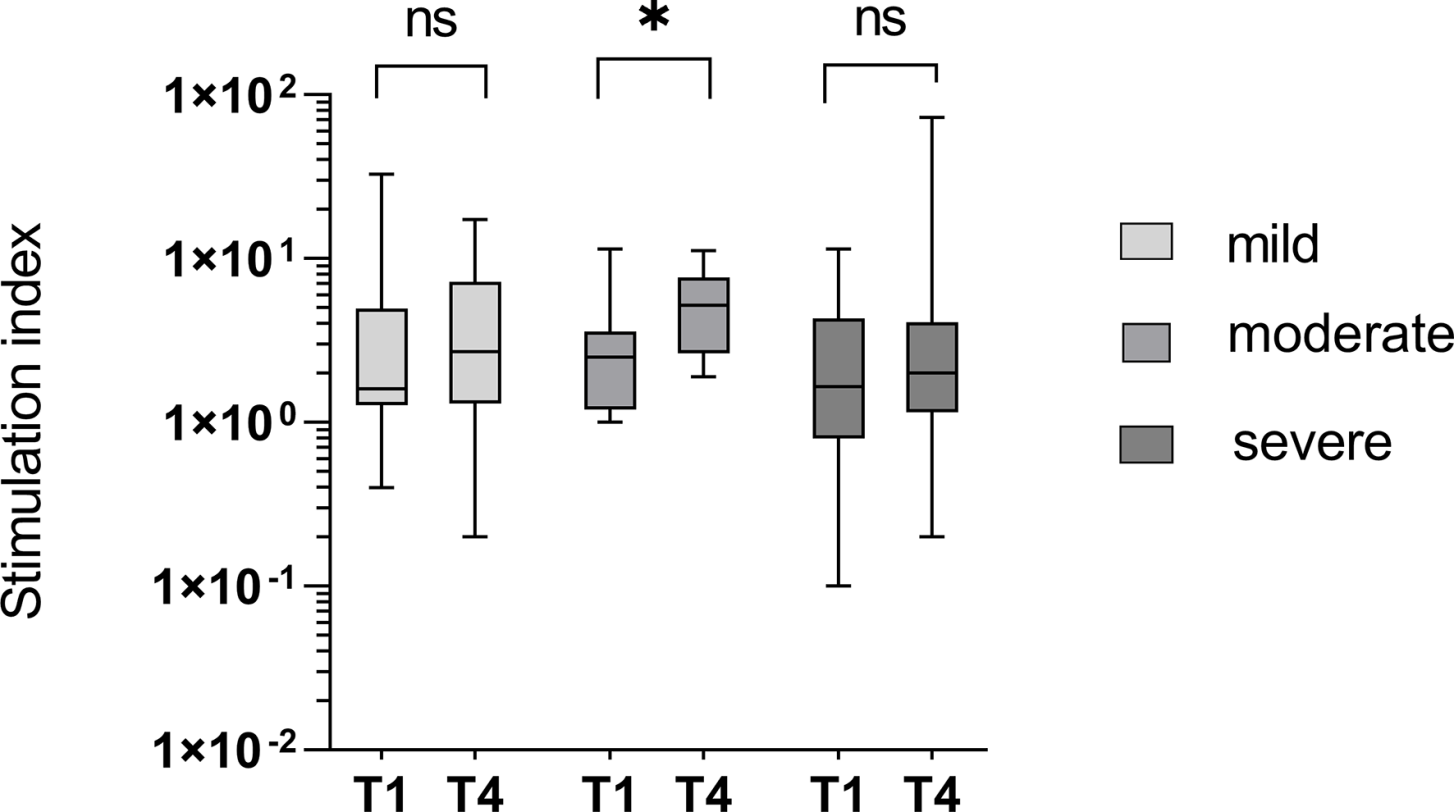
T-cell mediated immunity in groups divided into different courses of disease. An insignificant increase of IFN-γ-release from time point T1 to T4 was seen in patients with mild or severe courses of the disease (*p* = 0.962 and 0.794) and a significant increase in the study group with the moderate course (*p* = 0.038). ns - not significant; *p < 0.05.

At T1 there was no significant difference in the cellular immune response between patients suffering a subsequently mild, moderate, or severe course of infection (*p* = 0.735). The increase in the specific cellular response, measured by IFN-γ release in the ELISpot, was significantly higher in the patients with moderate courses compared to those with mild or severe courses (*p* = 0.038 versus *p* = 0.962 and 0.794, respectively), as shown in **Figure 9**. The stimulation index (SI) was 5.9 at T1 in the group with the mild course (n = 13) and decreased slightly to 4.8 within three months (p = 0.962). In the group with the moderate course (n = 13), the SI was 3.3 on average at T1 and increased significantly to 6.0 (p = 0.038). In the group of study participants with a severe course (n = 22), the SI developed from initially 3.6 to 6.7 (0.794).

### Development of neutralizing antibodies against SARS-CoV-2 WT, BA.1, and BA.5

Sera of 18 naïve and 39 imprinted study participants were investigated using plaque-reduction neutralization tests against the SARS-CoV-2 strains D614G, Omicron BA.1, and Omicron BA.5.

**Figure 10:**
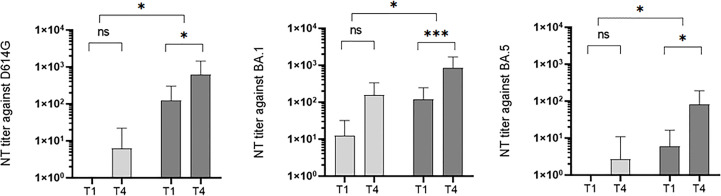
Development of neutralization titers against wildtype (WT), BA.1, and BA.5 over the study period at time points T1 (onset of the symptoms) and T4 (3 months after symptoms onset) in the two different study groups: naïve (n = 18) versus imprinted patients (n = 39) with SARS-CoV-2 Omicron BA.1 infection. ns - no significant difference; *p < 0.05; ***p > 0,001.

The initial antibody titer was significantly higher in the imprinted study group than in the naïve study group (0.4 versus 125.5 against D614G; 81.8 versus 118.2 against BA.1; 0.0 versus 5.8 against BA.5). The initial neutralizing titer in the naïve study group was highest when incubated with strain BA.1 (medium rank 81.8), and highest in the imprinted study group with the wildtype strain (medium rank 125.5). Interestingly, in contrast to the naïve study group, the neutralizing antibody titers increased significantly in the imprinted study group against all three strains tested (D614G, p = 0.4 vs 0.006; BA.1, p = 0.2 vs 0.000; and BA.5, p = 0.7 vs 0.02). Details in the development of neutralizing antibody titers in the two study groups of naïve versus imprinted BA.1 patients are shown in **Figure 10**.

## Discussion

In this study, we investigated the humoral and cellular immunological effects of infections with Omicron (B.1.1.529) BA.1 or BA.2 in infection-naïve versus (vaccine- or infection-) imprinted patients. Furthermore, we determined the neutralization capacity of the antibodies generated when infected with B.1.1.529 BA.1 or BA.2 in a neutralization assay with a new subvariant (B.1.1.529 BA.5). Finally, we discussed if immune imprinting by vaccination or previous infection with a former strain is an advantage or a disadvantage when infected with a future SARS-CoV-2 subvariant.

### Humoral immune response against B.1.1.529 in infection-naïve versus imprinted patients

Significant boosters of the specific humoral immune response were observed in both study groups. These significant boosters occurred in both groups within the first three weeks after symptom onset, but not before the end of the first week. These results are consistent with other study groups that found a boost in previous vaccine responses with Omicron breakthrough infections (15, 22). We did not observe a faster acquisition of the specific humoral immune response in our initially immunized study group, but the sampling time points of three months were not ideally chosen for this question, as seroconversion occurs faster and can generally be detected approximately 12 days after the onset of the symptoms (23).

### Cellular immune response against B.1.1.529 in infection-naïve versus imprinted patients

In our study, infection with Omicron B.1.1.529 led to an insignificant increase in the cellular-specific immune response in both study groups. This differs from the results of other research groups, who proved boosters of the cellular immune response in their naïve and vaccinated study groups (15). The time interval we chose between the ELISpots is longer than in other studies, so we might have overlooked short-lasting boosters as the specific IFN-γ response regulates itself somewhat downwards during this period. However, our findings are valid for the long-term effect on cellular immunity, which is not increased in imprinted patients.

Our studies on the cellular immune response to SARS-CoV-2 have shown that T-cell responses could be detected in some study participants who were not yet infected with the virus. This can probably be explained by cross-reactivity with related coronaviruses. Some studies showed that approximately 40-60% of infection-naïve individuals may show T-cell responses against SARS-CoV-2 (24–26), which is in line with our results.

A probable shortcoming of our study is that we could only test with peptide pools consisting of ancestral peptides of the regions S, N, M, and E and not against the Omicron-specific peptides of all of these regions. For specialization on Omicron, we only used the peptide pool consisting of Omicron-specific RBD (RBDo). Although intensive cellular specialization on RBD was not to be expected, since RBD is the main target of the humoral immune response and not the cellular one, we observed a significant reaction and increase in both study groups. This is consistent with other investigations using spike peptide mega pools, which have shown spike to be a possible main target of the cellular immune response (27–29).

### Neutralization capacity against a new subvariant in infection-naïve versus imprinted patients

We observed that infection with Omicron BA.1 or BA.2 produced comparable quantities of neutralizing antibodies against BA.1 in the PRNT. However, only the imprinted study group, in addition to neutralising BA.1, was also able to sufficiently neutralize D614G and BA.5 in the PRNT. Prior infection or vaccination history alters the antibody immunity and indeed imprints the immune response and leads to a more generalized antibody response. Presumably due to the previous significantly higher antibody titers, the neutralization capacity against sera of infection-imprinted BA.1/BA.2 patients was superior against the wild type, the infectious strain BA.1, and even against the emerging and currently predominant strain BA.5. In line with our results, a recent study showed that 30% of RBD WT-binding memory B cells in recipients of two doses of the BNT162b2 mRNA vaccine could be activated *in vitro* to secrete Omicron neutralizing IgG, suggesting broadly cross-reactive B cells (15, 22).

With all caution due to the limited number of participants, this can be interpreted as an indication that repeated challenges of the immune system with different genotypes contribute to a broader development of neutralizing antibodies and thus to better protection against future variants.

### Immune imprinting

With the emergence of new variants of SARS-CoV-2, there have been studies published that have addressed the issue of immune imprinting and found hints for ADE in a few patients´ sera (4). Immune imprinting is indeed described in some recent studies (30–32). Regarding SARS-CoV-2, this means that despite the booster with Omicron B.1.1.529 BA.1, the ancestral strain D614G is still better neutralized.

A closer investigation of the antibodies by immunoblotting revealed hints for moderate forms of immune imprinting: While the pe-imprinted study group showed a generalized immune response, the immunologically naïve group developed antibodies that were more specific for the current variant, as shown by an insignificantly higher increase in specific IgG antibodies against the Omicron-specific RBD in the naïve study group and the suppressed development of anti-RBDo antibodies in immunized study participants. The phenomenon of a more non-specific humoral immune response after triple vaccination and the formation of cross-reactive binding antibodies has also been described by other research groups (32, 31).

Through specification in the immunoblot, we were able to show further differences in antibody development between both groups. While the naïve study group mainly generated antibodies against RBD IgG, the imprinted study group mainly generated the already existing antibodies against different targets. An investigation of the effectiveness of the generated antibodies showed the high efficiency of the generally increased antibodies in the imprinted study group according to the motto “a lot goes a long way”.

### ADE

Diagnostic hints for antibody-dependent enhancement (ADE), such as the observation of increased severity of symptoms associated with early high levels of SARS-CoV-2 antibodies in patients, could not be observed. On the contrary, the study found that immune imprinting through vaccination or previous infection induces higher specific IgG titers, leading to better neutralization capacity even against a new variant. The major difference in humoral immune response between naïve and immune-imprinted patients was in the level of specific antibody titers, in favor of the immune-imprinted study group.

Additionally, we observed a significantly reduced increase of specific IgG antibodies in patients with severe outcomes and a moderate correlation between the course of the disease and the initial titer against the Omicron-specific RBD. Additionally, a moderate negative correlation between the course of the disease and the initial titer against N was observed, which suggests that the onset of infection and the order in which antibodies are developed at the onset of infection may have an impact on the outcome.

### The course of the disease in previous infection-naïve versus imprinted patients

The courses of the disease were shorter and the viral loads were lower in the group with breakthrough infections. However, neither the overall severity of Omicron infections nor the nasopharyngeal viral load was significantly different between either study group. This is in line with the results of other research studies, which generally diagnosed Omicron as having few severe courses (33, 34) despite higher transmissibility compared to the ancestral strain (35).

The strain type (Omicron BA.1 or BA.2) also did not affect disease progression. The generation of specific antibodies was generally reduced and the increase in the 3-month observation period was hesitant and significantly lower than in the comparison groups with mild to moderate courses. The cellular immune response also did not increase significantly in the group with the severe course of the disease. However, it must be said that we were also unable to determine a significant increase in the stimulation index in the specific IFN-γ ELISpot in the group with the mildly ill patients. There are distinct reasons for this difference: The insignificant increase in the specific cellular immune response in patients with a mild course of the disease can probably be attributed to a lower antigenic challenge (15) and the infection could be tackled mainly with the existing T cells combined with the humoral immune response. The combination of a weak humoral and cellular immune response, however, was attributed to a more severe course of the infection.

## Conclusion

Viral variant infection elicits variant-specific antibodies and prior mRNA vaccination or infection with a previous SARS-CoV-2 variant imprints serological responses toward the ancestral strain rather than variant antigens (31). On the other hand, our study shows that the initially higher specific antibody titers due to former imprinting *via* vaccination or prior infection significantly increased the humoral immune response. Imprinted study participants with a BA.1 or BA.2 infection developed a stronger humoral immune response with significantly higher antibody titers and significantly higher neutralizing abilities not only against the ancestral strain but also against the strain BA.1 and against the following predominant strain BA.5, and therefore outperform the humoral immune response of naïve study participants.

Preexisting immunity offers protection against infection with the same or another SARS-CoV-2 variant rather than a negative antibody-dependent enhancement. Naïve patients developed specific protection against the causative variant whereas imprinted patients developed antibodies against a broader spectrum of targets and potentially have better protection against future variants.

## Data availability statement

The datasets presented in this study can be found in online repositories. The names of the repository/repositories and accession number(s) can be found below: OQ520264 (Genbank).

## Ethics statement

The studies involving human participants were reviewed and approved by Ethics comitee of the Medical University of Innsbruck, no. 1064/2021. The patients/participants provided their written informed consent to participate in this study.

## Author contributions

STS: conceptualization, data curation, formal analysis, funding acquisition, investigation, methodology, and writing—original draft preparation; SW: investigation and methodology. LK: conceptualization, organization, methodology, validation, and verification. RP: patient recruitment; TT: investigation and methodology; EH: investigation, methodology, and writing—editing; SS: editing and funding acquisition; VM: project administration; WP: validation and supervision; GW: supervision, interpretation, discussion of clinical data and laboratory results, writing—original draft preparation, and editing. All authors contributed to the article and approved the submitted version.
