# Supplementary material for: Omicron (B.1.1.529) BA.1 or BA.2-related effects on immune responses in previously naïve versus imprinted individuals: immune imprinting as an advantage in the humoral immune response against novel variants
Source: Front Immunol. 2023 May 15;14:1165769. doi: 10.3389/fimmu.2023.1165769 (PMC10225645; doi:10.3389/fimmu.2023.1165769)
Supplement: Supplementary file 1 [file DataSheet_1.pdf]

APPENDIX

Table 3: Demographic data of the study participants, divided according to age group, gender, course of disease, vaccination status, vaccination type and initial serological status.

age group	n	%
21-30	6	10.2
31-40	15	25.4
41-50	9	15.3
51-60	15	25.4
61-70	9	15.3
71-80	5	8.5
total	59	100.0

gender	n	%
female	34	52.3
male	25	38.5
total	59	100.0

course of disease	n	%
mild	19	29.2
moderate	14	21.5
severe	26	40.0
total	59	100.0

vaccination status	n	%
vaccinated	41	69.5
unvaccinated	18	30.5
total	59	100.0

vaccination type	n	%
Astra	5	8.5
Biontech	20	33.9
Johnson	3	5.1
Moderna	10	16.9
partially vaccinated	3	5.1
unvaccinated	18	30.5
total	59	100.0

Omicron strain	n	%
BA.1	37	62.7
BA.2	17	28.8.
undefined	5	8.5
total	59	100.0

course of disease	naive	%	vaccinated	%	total
mild	7	11.9	12	20.3	19
moderate	2	3.4	12	20.3	14
severe	9	15.3	17	28.8	26
	18	30.5	41	69.5	59

initial serological status	n	%
seronegative	18	30.5
seropositive	41	69.5
total	59	100.0

Table 4: Study participants with the initial serological status (naïve versus imprinted) and their symptoms in the first week of infection with SARS-CoV-2 BA.1 or BA.2.

SeroStatus	No.	Symptoms in the first week of infection
naïve	LK 1	Temp 39.2°C, scratchy throat. Fever and flu symptoms, duration 3 days. Headache, feeling cold, night sweats, pressure in the ears, unproductive cough,
naïve	LK 2	Sore throat, headache and pain in the limbs, irritable cough. Purulent yellow coatings in the throat with swelling in the region of the lymph nodes on the larynx. afebrile. No expectoration.
naïve	LK 3	Temp. 40.7°C, pain in limbs, unproductive sporadic cough.
naïve	LK 4	Flu symptoms, 39.8°C, headache, aching limbs, nausea and palpitations, cold and pain in the ears, sporadic productive cough. Expectorate green-yellow colour. Apetite, sense of smell and taste intact.
naïve	LK 5	Temp.38,7°C, headache on NRS: 8. cough irritation. productive cough and headache NRS: 2-3.
naïve	LK 6	Headache on scale of 0-10: 10 and temp.37.5°C, stomach unwell, loss of appetite and general weakness and sporadic headaches. Sleep problems since onset of illness.
naïve	LK 7	Cold symptoms, afebrile.
naïve	LK 8	Cold symptoms, afebrile.
naïve	LK 9	Cold symptoms of throat and nose. Impaired sense of taste and smell: bitter, sour and partly sweet present.
naïve	LK 10	Onset of illness with temperature 37.8°C auricular, headache on NRS: 8, mild sore throat, swelling of lymph nodes on right side of throat: walnut-sized. Initially pressure-dolent.
naïve	LK 11	Onset of symptoms with sore throat, mild cough and severe limb pain, sporadic non-productive cough.
naïve	LK 12	Cold symptoms, worsening, headache on scale 8, continuous aching limbs, sporadic nosebleeds, productive cough. Sense of smell intact.
naïve	LK 13	Headache with worsening, productive cough, expectorate yellowish colour, subfebrile.
naïve	LK 14	Mild cold symptoms
naïve	LK 15	Cold symptoms
naïve	LK 16	Cold symptoms, productive cough
naïve	LK 17	Cold symptoms, general weakness.
naïve	LK 18	Pain in the back, neck, upper arms, chest, head and behind ears. Dizziness, sore throat, cough and cold symptoms.
naïve	LK 19	Headache on scale: 4, hoarseness, increased unpleasant sensitivity, increased sweating on exertion and weakness.
naïve	LK 20	Weakness, continuous headache on scale 2- 8, worsening in the evening," flu-like" feeling, sore throat, swelling of the lymph nodes on the right, diarrhoea until evening, half-hourly. Pulling in the right ear. Now still productive cough and irritable cough, especially at night. Increased feeling of thirst (drinks up to 6 litres per day).
naïve	LK 21	Loss of appetite, hypersensitivity, especially in the arms and legs and at night. Temp. 38.5°C, altered sense of smell and taste.
naïve	LK 22	Chills and cold symptoms
naïve	LK 23	Cold symptoms, afebrile.
naïve	LK 24	Sore throat and productive cough.
naïve	LK 25	Temp. 38.5°C, headache
naïve	LK 26	Headache for 2 days, fatigue, aching limbs
naïve	LK 27	Rhinitis, irritable cough and productive cough.
naïve	LK 28	Sore throat, temp.37.9°C axillary, pressure in the head
imprinted	LK 29	Headache on scale 9, sporadic cough, sporadic dizziness and fatigue with physical activity.

imprinted	LK 30	Mild cold symptoms
imprinted	LK 31	Feeling of sickness, lack of appetite, weakness, temp. 39.0 °C.
imprinted	LK 32	Sore throat on NRS: 8.
imprinted	LK 33	Sore throat on NRS: 8. temp 39.2°C.
imprinted	LK 34	Mild cold symptoms
imprinted	LK 35	Cold symptoms
imprinted	LK 36	Cold symptoms, productive cough, mild fever
imprinted	LK 37	Back pain in the lumbar region and hip pain bilaterally, Remained constant on NRS 3-4, Cold symptoms and sore throat and headaches.
imprinted	LK 38	Sweating, headache, intermittent dizziness, herpes labialis.
imprinted	LK 39	Sore throat and headache
imprinted	LK 40	Cold symptoms, temp. 39.2°C, sense of smell impaired.
imprinted	LK 41	Feeling hot and cold and flu symptoms.
imprinted	LK 42	Headache and earache. Discomfort in the nose. intermittent headaches.
imprinted	LK 43	Cold symptoms
imprinted	LK 44	Temp. 38.9°C. Headache NRS 8. Productive cough. Salty taste diminished.
imprinted	LK 45	Vomiting 1x and bedriddenness. Absence of sense of smell and taste.
imprinted	LK 46	Weakness. Impaired sense of smell and taste. Irritative cough and productive cough. Temp. approx. 38.0°C.
imprinted	LK 47	Cold symptoms. Loss of appetite. Fatigue, productive cough. Slightly impaired sense of smell and taste.
imprinted	LK 48	Cold symptoms. Flu symptoms. Headache on NRS 8-9. Feeling of cold. Temp. 37.5°C. Nausea. Lack of appetite and cough.
imprinted	LK 49	Cold symptoms
imprinted	LK 50	Cold symptoms
imprinted	LK 51	[illegible] painless.
imprinted	LK 52	Chills and 38.9°C temperature. Rhinitis, eczema on back of hands without itching, poor quality of sleep at night.
imprinted	LK 53	Cold (sore throat and loss of voice) for 3 weeks. Headache NRS 9, sore throat, feeling hot and cold.
imprinted	LK 54	Cold symptoms
imprinted	LK 55	Cold symptoms
imprinted	LK 56	Cold symptoms with fever
imprinted	LK 57	Cold symptoms
imprinted	LK 58	Cold symptoms, non-productive cough, snuffy.
imprinted	LK 59	Cold symptoms, sporadic productive cough, greenish secretion.
